# Wounds of the Brain

**Published:** 1889-11-30

**Authors:** 


### SURGERY.

### WOUNDS OF THE BRAIN.

Dr. G. Archie Stockwell, of Port Hurun, Michigan, has some observations in a recent *Philadelphia Therapeutic Gazette* on brain wounds with excessive loss of structure. Several cases of injury are recorded when a considerable amount of brain matter was lost, in one case "not less than six or eight heaping tablespoonfuls of brains." The person the subject of this loss afterwards had a hernia cerebri. But on recovery he abandoned his former occupation and became a school teacher, subsequently blossoming as a phrenological lecturer. "He recently died in or near the Grand Rapids in Michigan, and the only mental aberration ever detected was a decided disinclination to live honestly or to meet his lawful debts and obligations, the bill for medical attendance included." Extensive loss of brain substance without apparent ill effects is not by any means novel. Erichsen states that children especially bear the loss of a portion of cerebral matter without any ill effect. And authors on military surgery have recorded various instances of the kind in adults. For instance, Hennen tells us that by sabre cuts inflicted by our own and the French dragoons in Spain and Belgium, sections of the scalp, cranium, and brain itself were frequently made, and in some cases successfully treated. Guthrie in his commentaries on the surgery of the Peninsula War notices several cases. McLeod, in "Notices of the Surgery of the Wars in the Crimea," details a case, and the latter author tell us that in injuries of the brain coming under his notice the symptoms were seldom in accordance with the amount of injury. It is on record that twenty French soldiers, whose vertices, with more or less of the brain, were cut off by sabre strokes, had not at first a single bad symptom, and performed a journey of forty leagues, one half the distance being travelled on foot. Some time ago Mr. Bryant brought a case before one of the London societies of a man who was struck on the forehead, just above the nose, whilst looking out of the window of a railway carriage. The skull was fractured, and portions of the brain protruded. Yet the individual was able to walk to the station and complain. It would appear that wounds of the anterior and upper portions of the brain are least dangerous. Although, however, so many cases of recovery after loss of brain matter are recorded, they are, nevertheless, too rare to be dwelt upon as holding out hope in bad cases.

